# Efficacy and safety of Re-Du-Ning injection in the treatment of seasonal influenza: results from a randomized, double-blinded, multicenter, oseltamivir-controlled trial

**DOI:** 10.18632/oncotarget.19220

**Published:** 2017-07-13

**Authors:** Yu Liu, Wei Mu, Wei Xiao, Bao-Lin Wei, Lan Wang, Xin-Qiao Liu, Xu-Dong Xiong, Xiao-Min Huang, Ye-Qing Zhang, Hai-Ming Chen, Feng-Jie Yan, Yu-Ping Tan, Yu-Hong Huang

**Affiliations:** ^1^ Shenzhen Traditional Chinese Medicine Hospital Affiliated to Guangzhou University of Traditional Chinese Medicine, Shenzhen 518033, China; ^2^ The Second Affiliated Hospital of Tianjin University of Traditional Chinese Medicine, Tianjin 300150, China; ^3^ State Key Laboratory of New-tech for Chinese Medicine Pharmaceutical Process, Jiangsu 222001, China; ^4^ Dongzhimen Hospital Affiliated to Beijing University of Traditional Chinese Medicine, Beijing 100007, China; ^5^ The First Affiliated Hospital of Tianjin University of Traditional Chinese Medicine, Tianjin 300193, China; ^6^ Shanghai ShuGuang Hospital Affiliated to Shanghai University of Traditional Chinese Medicine, Shanghai 201203, China; ^7^ Zhejiang Hospital of Traditional Chinese Medicine, Hangzhou 310006, China; ^8^ Jiangsu Hospital of Integrated Traditional Chinese and Western Medicine, Nanjing 210028, China; ^9^ Affiliated Hospital of Liaoning University of Traditional Chinese Medicine, Shenyang 110032, China; ^10^ Affiliated Hospital of Changchun University of Traditional Chinese Medicine, Changchun 130021, China; ^11^ Ruikang Hospital Affiliated to Guangxi University of Chinese Medicine, Nanning 530011, China

**Keywords:** Re-Du-Ning injection, seasonal influenza, oseltamivir, clinical trial

## Abstract

**Objective:**

To assess the efficacy and safety of RDNI in the treatment of seasonal influenza.

**Results:**

1575 participants were screened and 229 completed the study and had a RT-PCR laboratory confirmation of influenza virus infection. Fever alleviation time was 2 and 6 hours, and fever clearance time was 27 and 47 in RDNI and oseltamivir, with significant difference between two groups. Total scores of influenza symptoms descended more in RDNI than oseltamivir on day 2 and day 3. Single symptom such as fever, aversion to cold, sore throat and nasal obstruction score descended more in RDNI than oseltamivir on different days. 20 subjects used aspirin during the trial, and there was no significant difference between two groups.

**Materials and Methods:**

We conducted a randomized, double-blind, double-dummy, oseltamivir controlled clinical trial. Patients with a positive influenza rapid test diagnosis were enrolled and randomized to receive RDNI or oseltamivir. Primary outcome was the median fever alleviation and clearance time. Secondary outcomes were total 8 influenza symptom scores, the single influenza symptom score, and the frequency of aspirin usage.

**Conclusions:**

The effect of RDNI was not worse than oseltamivir on the alleviation of influenza symptoms. RDNI was well tolerated, with no serious adverse events noted during the study period.

## INTRODUCTION

### The influenza epidemic

According to WHO reports, Influenza is an acute respiratory infections disease which can result in annual infection of 5–15% of the population, leading to 250,000 and 500,000 deaths [1]. Numerous outbreaks of influenza/Influenza-like (ILI) illness are reported every year throughout China. From April 1, 2005 to November 30, 2013, there were 2.768 influenza/ILI outbreaks recorded in the Emergency Public Reporting System. Influenza is caused by type A, including H1N1, H3N2, H5N1, H7N9, and type B influenza viruses. Such respiratory disease is characterized by sudden onset of high fever, aversion of cold, headache, myalgia, sore throat, fatigue and cough [2].

### Antiviral treatment drug

Antiviral treatment is by far the second most effective approach to influenza management apart from vaccination.

At present, the anti-influenza virus drugs already on the market are divided into two classes, one is M2 ion channel blockers represented by amantadine and rimantadine, the other is neuraminidase (NA) inhibitors represented by Oseltamivir, zanamivir, and peramivir [3]. Though the protection rate of amantadine and rimantadine can reach 61% [4], which is done through inhibiting the replication of influenza A virus, amantadine and rimantadine have no effect against influenza B virus and can cause serious gastrointestinal adverse reactions and central nervous system side effects [4]. In addition, it has been found that influenza A virus produces resistance to M2 ion channel blockers because of overuse [5]. Even countries where these medicines have not been widely used occurred M2-resistant variants [6]. Pandemic 2009 has demonstrated a high level of resistance to amantadine and rimantadine [7, 8]. In Guangzhou of China, the clinical separation of H1N1 and H3N2 occurs adamantane resistance up to 93% and 100%, respectively [9].

Compared with M2 ion channel blockers, NA inhibitors are more widely used, but they are also associated with gastrointestinal adverse effects [10], rare mental system severe side effects [11] and respiratory side effects [12]. Moreover, NA inhibitors may produce less resistance in the case of widespread use. A worldwide survey between 2004 and 2008 reported that oseltamivir-resistance is very low level (< 1%) [13], but other surveys from 2007 to 2009 showed the resistance oseltamivir-resistance to be more than 90% [14–16]. Zanamivir resistance has also been reported in immunocompromised people [17–18].

### Traditional Chinese medicine

Traditional Chinese medicine has a long history of being used in the treatment of respiratory infection disease. TCM has the characteristics of multi-target treatment and, potentially, may help to avoid the emergence of drug resistance and side effects. So, it may be a good choice to export the new antiviral medicine from TCM. Traditional Chinese Medicine injection is a kind of new TCM preparations and was mainly used for treatment of acute and severe disease. Among these TCM injections, Re-Du-Ning injection (RDNI), approved by CFDA (China Food and Drug Administration) in May 2005 in the treatment of upper respiratory tract infection, has been wide used in China. RDNI was highlighted as a recommended drug for the treatment of H7N9 influenza infection in *2014 diagnosis and treatment plan of H7N9* in China. In addition, RDNI has been prescribed to nearly 20million patients in China until now, but the incidence of adverse reactions was only 0.4%, mainly skin itching.

**Figure 1:**
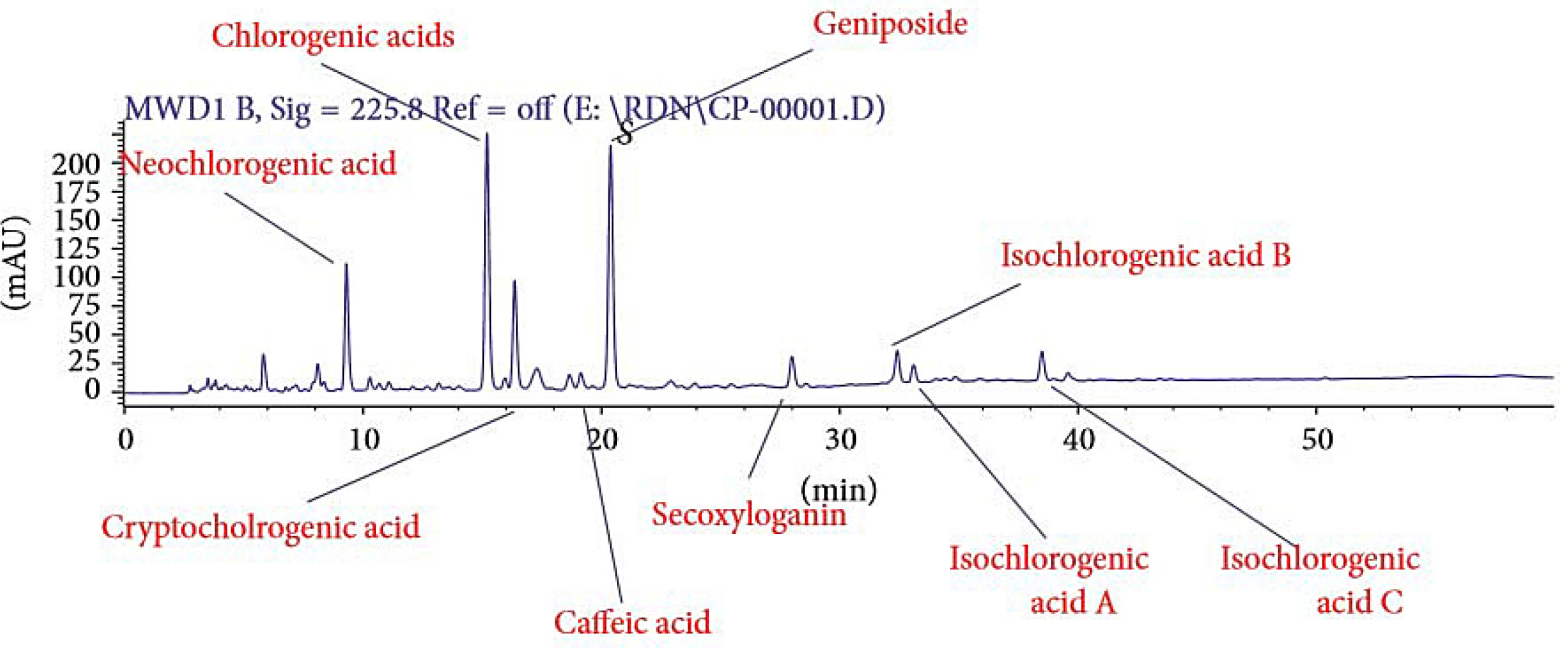
Liquid quantitative fingerprint of Re-Du-Ning injection

RDNI is composed by three herbal, namely *sweet wormwood herb, fructus gardeniae, honeysuckle*. The extracts of these three herbal components can inhibit respiratory syncytial virus, adenovirus, herpes simplex virus, parainfluenza virus, and influenza virus *in vivo* and vitro [19–21]. Nine ingredients of RDNI were listed in finger-print for quality control in Figure 1. Many studies have also verified standardized RDNI has antipyretic, anti-inflamatory, analgesic bioactivity, and anti-influenza viral (H1N1,H3N2, influenza B) effects [22–25].

### Study aims

Although many studies have suggested RDNI may inhibit the influenza virus, there is no clinical trials confirm the inference, except our previous small-sample clinical study [26], which evaluated the efficacy of RDNI in the treatment of 34 etiology diagnosed influenza patients. Our previous study indicated that RDNI may be more effective than Oseltamivir in alleviating fever and easing influenza symptoms. We prefer to do a further evaluation of the efficacy and safety of RDNI in the treatment of seasonal influenza in a larger sample size clinical trial.

## RESULTS

### Participant enrollment flow

**Figure 2:**
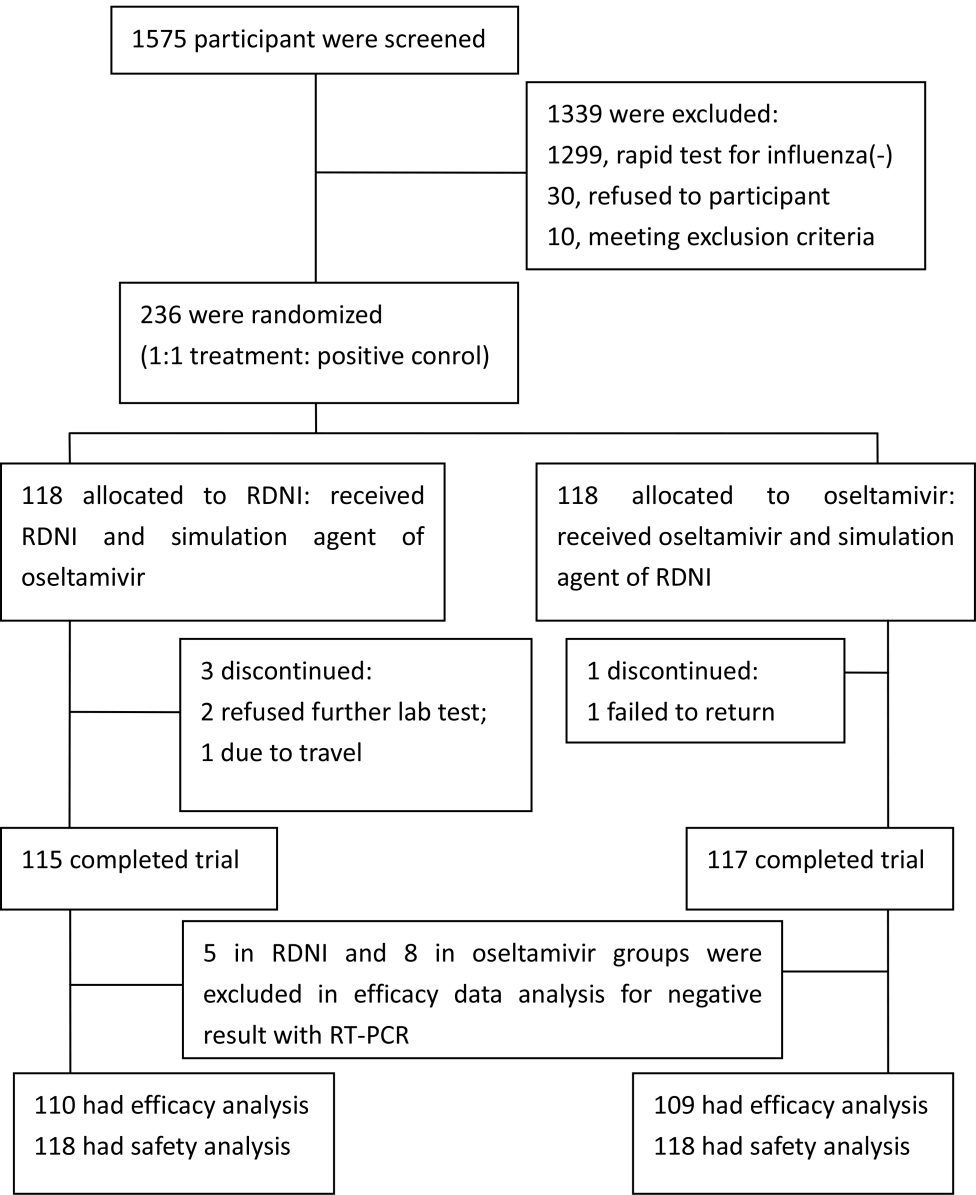
Participant flowchart

1575 patients from 9 clinical centers were screened. Of them, influenza rapid test of 276 (18.00%) were positive. After further screening, 236 participants were included and randomly assigned to RDNI group (*n* = 118) and Oseltamivir group (*n* = 118). 232 participants completed the study (115 in RDNI group and 117 in Oseltamivir group). Among them, 5 in RDNI and 8 in Oseltamivir group had negative result with RT-PCR, who were excluded in efficacy evaluation. (Figure 2)

### Demographics and clinical characteristics at baseline

Table 1 shows the baseline characteristics of included participants. No significant differences of demographic characteristics, in addition to sex ratio were observed between RDNI and Oseltamivir groups. More females were included in Oseltamivir group than in RDNI group (68.64% v.s. 52.54%), the difference was statistically significant (*p* = 0.0163). At baseline, axillary temperature, course of disease, total symptom scores and every single symptom score were equivalent in both groups. (Table 1).

**Table 1:** Demographic and clinical characteristics of the participants at the baseline

Characteristic	Study groups	
	RDNI (*n* = 118)	Oseltamivir (*n* = 118)
**Demographics**		
Age, mean (SD), y	37.97 (13.87)	36.89 (13.85)
Men, number (%)	56 (47.46)	37 (31.36)
Drug used before enrollment, number (%)	3 (2.54%)	2 (1.69%)
Co-existing disease, number (%)	8 (6.78%)	6 (5.08%)
Combined medicine, number (%)	3 (2.54%)	2 (1.69%)
Course of disease, M(Q), h	10.00 (4.00)	11.50 (5.00)
**Outcome measures at baseline**		
Axillary temperature, mean (SD),°C	38.95 (0.28)	38.94 (0.27)
Total symptom scores, mean (SD)	19.74 (4.84)	20.40 (4.96)
Single ‘fever’ symptom score, mean (SD)	7.63 (1.50)	7.55 (1.51)
Single ‘aversion to cold’ symptom score, mean (SD)	2.90 (1.35)	2.93 (1.38)
Single ‘myalgia’ symptom score, mean (SD)	2.39 (1.18)	2.68 (1.41)
Single ‘Cough’ symptom score, mean (SD)	1.51 (0.84)	1.66 (0.85)
Single ‘headache’ symptom score, mean (SD)	1.28 (0.85)	1.38 (0.84)
Single ‘sore throat’ symptom score, mean (SD)	1.58 (0.90)	1.65 (0.87)
Single ‘fatigue’ symptom score, mean (SD)	1.38 (0.90)	1.44 (0.86)
Single ‘nasal obstruction’ symptom score, mean (SD)	1.07 (0.89)	1.10 (0.81)
**Infected, number (%)**	113 (96.00)	110 (93.00)
Influenza A/H1N1, number	48	46
Influenza A/H3N2, number	21	26
Influenza A/uc^§^, number	12	7
Influenza B, number	32	31

^§^uc: unclear.

### Primary outcomes

Among influenza-infected participants, the median fever clearance time was 27 and 47 hours (P50) in RDNI and Oseltamivir groups, respectively, and the median fever alleviation time was 2 and 6 hours, respectively. The HR (hazard ratio) of fever clearance time was 0.477 (95% CI was from 0.36 to 0.62). The HR of fever alleviation time was 0.345 (95% CI was from 0.25 to 0.46). HR of both primary outcomes were less than one(RDNI group/Oseltamivir group), and there were statistical differences (*p* < 0.0001), indicating that relief and abatement of fever was faster in RDNI group than in Oseltamivir group (Table 2).

**Table 2:** Duration of fever symptom in RDNI and oseltamivir groups

	Influenza-infected participants		All treated paticipants	
	RDNI (*n* = 110)	Oseltamivir (*n* = 109)	RDNI (*n* = 118)	Oseltamivir (*n* = 118)
Fever clearance time, *P*_50_(*P*_25_˜*P*_75_), h^※^	27 (25˜28)	47 (45˜48)	27 (25.5˜28.5)	47 (45˜48)
HR, (95%CI)/*P*	0.477 (0.362˜0.628)/<0.0001^※^		0.479 (0.367˜0.626)/<0.000^#^	
Fever alleviation time, *P*_50_(*P*_25_˜*P*_75_), h^※^	2 (1.5˜2)	6 (/˜/)	2 (1.5˜2)	6 (/˜/)
HR, (95%CI)/*P*	0.345 (0.258˜0.460)/< 0.0001^#^		0.335 (0.254˜0.443)/< 0.0001^#^	

^※^*P*_50_ indicates the median time. *P*25 and *P*75 indicate lower quartile and upper quartile. #HR< 1, and *P* < 0.0001 indicates that fever alleviation and clearance time are less in RDNI than in Oseltamivir, and with statistical differences.

In clinical practice, since medicine to treat influenza always was used without confirmed influenza infection using PCR or viral culture, we also performed an analysis of the effect on all participants regardless of microbiologic results. Similarly as the result of influenza-infected participants, individuals receiving RDNI returned to normal body temperature more rapidly than individuals receiving Oseltamivir (Table 2).

### Sencondary outcomes

### Total influenza symptoms score

Compared with before treatment (baseline), both RDNI and Oseltamivir groups reduced the total symptoms score significantly on day 2, day 3, day 4 and day 6 (*P* < 0.0001). Total symptoms score descended significantly in RDNI group more than in Oseltamivir group on day2 and day 3 (*P* < 0.05). The decline of total symptoms score on day 2 and day 3 was 9.31 ± 4.41 and 14.96 ± 5.80 in RDNI group, respectively, 7.53 ± 4.75 and 12.85 ± 6.82 in Oseltamivir group. No significant difference was shown on day4 and day6 between two groups (Table 3).

**Table 3:** Decline of total symptoms scores in the group itself and between two groups

Time	Group	N	PO-PR $\bar{X}$ ± s (95%CI)	Within-group		T-C $\bar{X}$ ± s (95%CI)	Between-group	
				*t*-test	*P*-value		F	*P*-value
Day 2	T	118	9.31 ± 4.41 (8.51, 10.12)	22.94	< 0.0001	−1.78 ± 0.60 (0.60, 2.96)	9.5	0.002^#^
	C	118	7.53 ± 4.75 (6.67, 8.40)	17.21	< 0.0001			
Day 3	T	118	14.96 ± 5.80 (13.90, 16.02)	28.00	< 0.0001	−2.11 ± 0.82 (0.49, 3.73)	6.94	0.009^#^
	C	118	12.85 ± 6.82 (11.60, 14.09)	20.47	< 0.0001			
Day 4	T	118	16.60 ± 5.51 (15.60, 17.61)	32.72	< 0.0001 −1.02 ± 0.78	(−0.53, 2.56)	1.99	0.160
	C	118	15.58 ± 6.47 (14.40, 16.76)	26.15	< 0.0001			
Day 6	T	118	17.65±5.83 (16.59,18.71)	32.92	< 0.0001 −0.44 ± 0.84	(−1.21, 2.09)	0.36	0.547
	C	118	17.21±6.98 (15.94,18.48)	26.79	< 0.0001			

T: treatment group (RDNI); C:controlgroup(Oseltamivir); PO−PR, post treatment score minus prior treatment score; $\bar{X}$ ± s: Mean ± SD; T−C: treatment group score minus control group score, and ^#^significant difference between groups.

### Single symptom score

**Figure 3:**
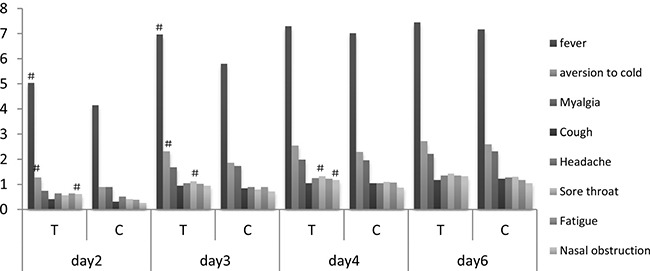
Decline of single symptom severity score T, treatment group (RDNI); C, control group (oseltamivir); ^#^between two groups, comparing with control group, fever score and aversion to cold score of RDNI descended significantly on day 2 and day 3 (*P* < 0.01); sore throat score of RDNI descended significantly on day 3 and day 4 (*P* < 0.05); nasal obstruction score of RDNI descended significantly on day 2 and day 4 (*P* < 0.05). Within the group itself, comparing with prior treatment score, post treatment score descended significantly on day 2, day 3, day 4 and day 6 (*P* < 0.0001).

Alleviation of single symptom was similar to the downward trend of total influenza symptoms score. RDNI and Oseltamivir groups reduced every symptom score significantly from day 2 to day 6 (*P* < 0.0001), compared with before treatment. Meanwhile, significant difference was observed in fever, aversion to cold, sore throat and nasal obstruction score between two groups on different days, these single symptoms score descended more in RDNI group than in Oseltamivir group (Figure 3).

### Frequency usage of aspirin for alleviating fever

Because axillary temperature were above 39°C for 4 hours after given study medication, 20 participants used aspirin or aspirin effervescent tablets during the trial, 12 in RDNI group, 8 in Oseltamivir group. All of them had laboratory confirmed influenza infection. No statistically significant difference were identified between two groups. (Table 4).

**Table 4:** Treatment drug for alleviating fever used in RDNI and Oseltamivir groups

Whether apply other drug for alleviating fever?	Groups		*X*^2^	*P*
	RDNI *n* = 118	Oseltamivir *n* = 118		
Yes, *n* (%)	12^#^(10.17)	8^※※^(6.78)	0.874	0.350
No, *n* (%)	106 (89.83)	110 (93.22)		

^※^4 participants used compound aspirin tablets, the other 4 participants used aspirin effervescent tablets.

^#^4 participants used compound aspirin tablets, the other 8 participants used aspirin effervescent tablets.

### Adverse event

Though there are 7 adverse events in the trial, no participants withdrew (Figure 2). Hepatic transaminases increased slightly in 4 participants (2 in Oseltamivir and 2 in RDNI group), and 3 cases in Oseltamivir group may be related to the test drug. 1 participant had mild vomiting and recovered spontaneously. Blood leukocytes of 1 participant descended slightly after treatment. Red blood cells of 1 participant increased slightly after treatment. In the study, no serious drug-related adverse events occurred and there was no use of rescue medication.

## DISCUSSION

Despite some nature herbal medicine have been already used to treat influenza (H1N1) in China [27], the evidence to complementary treatments for influenza is limited. RDNI is a combination of 9 ingredients extracted from *sweet wormwood herb, fructus gardeniae, honeysuckle*. RDNI being used for treating upper respiratory tract infection 11 years in China, recently has been administered as an alternative therapy against seasonal influenza in some influenza outbreaks. We conducted the multicenter, randomized, double-blinded, and oseltamivir-controlled study to evaluate the efficacy and safety of RDNI in the treatment of influenza patients.

Different from other trials [28], considering that specimen transportion and confirmed infection using RT-PCR usually took a long time, to ensure participants received treatment within 48 h of the illness onset and to improve compliance of participants, the throat rapid test was used to screen the potential eligible patients. Later on, we compared influenza-infected and all-treated subjects in statistical analysis.

Influenza is a self-limiting disease, whose clinical features are aversion of cold, myalgia, cough, headache, sore throat, fatigue, nasal obstruction, symptoms disappearance and life quality improvement are pursued. Therefore, in our study the outcomes focused on the relief of these symptoms.

In this study statistically significant difference was observed in the fever alleviation and clearance time between two groups, not only in influenza-infection and but also in all treated subjects. Both RDNI and oseltamivir effectively shortened the duration of influenza fever (P50 = 2 v.s. 6 hours for fever clearance time; P50 = 27 v.s. 47 hours for fever alleviation time) and CHDI outperformed oseltamivir (HR = 0.345 for fever alleviation time and HR = 0.479 for fever clearance time, *P* < 0.0001). It seems worthwhile to state that, in our previous small-sample study which enrolled 46 patients (34 cases were confirmed influenza virus infection), though there is trend that the fever alleviation time and clearance time of RDNI are less than Oseltamivir group (P50 = 2.5 v.s. 5 hours for fever clearance time; P50 = 32.5 v.s. 49 hours for fever alleviation time), no statistical difference of HR has been shown.

Our previous study found that both RDNI and Oseltamivir treatment can significantly alleviating influenza symptoms score, otherwise, RDNI have better effect than Oseltamivir in reliving fever on day 2. In the present study, this result was further confirmed. The decline of total symptoms scores was more significantly in RDNI than in Oseltamivir not only on day 2, but also on day 3. Meanwhile, RDNI also showed a significant reduction than Oseltamivir in aversion to cold score, sore throat score and nasal obstruction score.

Differently from our previous study, infusion reaction of RDNI was not observed in the present study. In this trial 2 participants in RDNI group showed a rise in ALT and GGT respectively (ALT = 60 IU/L v.s. 76 IU/L at baseline and after treatment; GGT = 69 IU/L v.s. 120.8 IU/L at baseline and after treatment), and recovered normal within one week after the trial. There is no serious drug-related adverse events. Overall, RDNI and Oseltamivir were well tolerated in this study.

Though our study is a randomized, double-blind, large-sample and multi-center clinical trial, and the majority of included patients (95.3%) had confirmed laboratory diagnosis of influenza virus infection, some shortcomings in the study should be noted. First, considering influenza can cause severe viral pneumonia, which occur even in healthy young adults, and many secondary influenza complications, our study did not use the placebo as control. Second, patients with severe concomitant diseases and some special population, such as elderly and children were excluded in our study. Third, this study protocol only included clinical symptom as outcome measure, virus replication employing RT-PCR has not been assessed as an outcome during treatment, which is commonly used in other influenza clinical trials [28, 29]. Fourth, our study did not addressed economic evaluation, such as lost time from work, which is an important effect of influenza, especially on healthy people [30].

In summary, this study demonstrated that RDNI had a significant effect on the alleviation of fever, cough, sore throat, headache, fatigue, nasal obstruction, myalgia and aversion to cold.

## MATERIALS AND METHODS

Ethical approval of the study protocol was granted from the Ethics Committee of the Second Affiliated Hospital of Tianjin University of Traditional Chinese Medicine, and informed consent was obtained from each participant. The registration number is ChiCTR-TRC-13004045.

### Design

**Figure 4:**
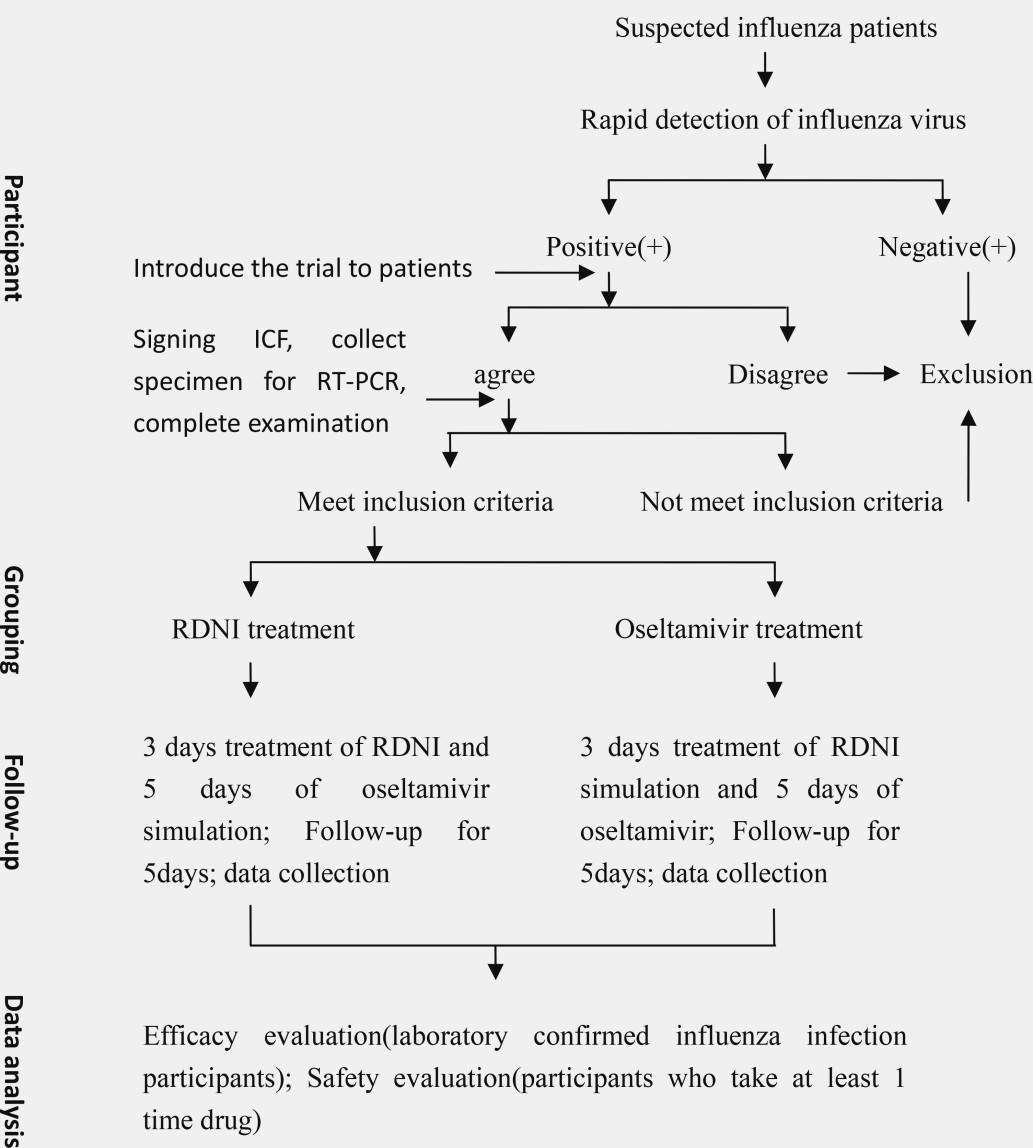
Participants with a rapid diagnosis of influenza will be recruited and randomized into two different treatment groups After 5 days of treatment and follow-up, efficacy and safety of the treatment were evaluated.

This clinical study was a prospective, multicentre, double-blinded, double-dummy, randomized trial. Non-inferiority trial, versus positive control, was conducted. Patients were enrolled from 9 clinical trial centers in 8 cities across mainland China, from Jan. 2014 to Mar. 2014, when the epidemic of influenza in China was reported by Chinese Center for Disease Control and Prevention. Patients with positive throat rapid test of antigens to the influenza virus (if detection of nucleic acid of the influenza virus was negative, the patient will be excluded in efficacy data analysis after the treatment) were assigned randomly into two groups of equal proportions. Groups were: Oseltamivir treatment (positive control) and RDNI treatment (test group). Follow-up was undertaken on baseline, 1 day and 2 days, and 3 days and 5 days after treatment. (Figure 4).

### Setting

Different aspects of the study activities were conducted at different sites. Patients enrollment, biochemical examinations, collection of specimens and data, were finished in 9 clinical trial centers. mRNA of influenza virus detecting were carried out in the National Influenza Centre (NIC), Beijing, China. Data management and analyses were done at Nanjing Medical University, Nanjing, China.

### Sample size

The study was powered as a non-inferiority study, and the primary endpoint was the fever clearance time. Non-inferiority boundary was 4 hours between two groups. Sample size was based on combined standard deviation of 10.26 (calculated on the basis of our previous clinical study [26]), with an error probability of 0.025 (single-tailed alpha error) and 0.2 for beta, computation formula described following yielded a sample size of 103 subjects per treatment arm. Considering a possible 20% expulsion rate, the designed sample size should be 120 per treatment arm.

 Computation formula and process

 N = 2[(Z1−a + Z1−β)(S/δ)]2

 Z1−a = Z1−0.025 = Z0.975 = 1.960

 Z1−β = Z1−0.2 = Z0.80 = 0.845

 N = 2[(1.960 + 0.845)(10.26/4)]2 = 2[2.805 × 10.26 ÷ 4]2 = 2 × 7.192 = 103

### Laboratory virus testing

Posterior pharyngeal throat swabs for isolation of influenza virus were taken at baseline. Swabs were taken from enrolled patients’ throat, placed into 3 mL of viral transport medium, and transported at 4°C by special courier to the NIC for further confirmation of influenza virus infection and subtype identification using RT-PCR analysis and a standard hemagglutination inhibition assay.

### Study procedure

### Inclusion and exclusion criteria

The inclusion criteria were: previously healthy adults aged 18 to 65 y; onset of influenza symptoms within 48 hours; axillary temperature that was 38.5°C or higher; at least one or more respiratory symptoms (sore throat, cough, and nasal congestion); at least one or more general symptoms (headache, fatigue, and myalgia); a positive throat rapid test for influenza performed by the practitioner using influenza virus antigen detection kit.

The exclusion criteria were: receiving influenza vaccination 12 months prior to the screening; routine blood WBC greater than the upper limit of normal value; having chronic respiratory diseases or pneumonia; having clinically significant chronic illness or human immunodeficiency virus disease; receiving systemic steroids or other immunosuppressants 3 months ago; pregnant women.

### Drugs and usage

### Re-Du-Ning treatment group

Patients received RDNI plus simulation agent of Oseltamivir. RDNI (20 mL) was added in normal saline 250 mL and intravenously administered by research nurses, once a day for 3 days. Oseltamivir simulation were administered 75 mg (75 mg/per capsule orally), twice a day for 5 days.

### Oseltamivir positive control group

Patients received Oseltamivir plus simulation agent of RDNI. RDNI simulation was saline 250 mL. The medicine preparation and administration of this group was the same with of treatment group.

### Location of drug usage

On treatment days 1, 2, and 3, in addition to take Oseltamivir or its simulation, patients received RDNI or its matching placebo at hospital. On treatment days 4 and 5, they took Oseltamivir or its simulation agent at home. On days 6 (after treatment), they got back to hospital for follow up examination.

### Other treatment

Four hours after the first time use of study medication, patients would be instructed to take aspirin if their axillary temperature was still above 39°C. The use of aspirin and any other medications was recorded in patient dairy card. Compliance was assessed by checking patient records of the date and time of each dose and verified by counting capsule returned by each patient.

### Randomization and blinding

### Randomization

Patients were randomized according to a predefined computer-generated randomization list with the balanced 1:1 randomization using a block size of four. A research pharmacist at Nanjing Medical University received the study medication from the producer of RDNI, Jiangsu Kangyuan pharmaceutical co., LTD., prepared the study medication according to the randomization schedule and then shipped study medication to 9 clinical sites, which distributed the numbered container of study medication to research nurses sequentially, when eligible participant was enrolled.

### Blinding

Because the color of RDNI is light yellow, the brown infusion tube was applied in infusion operation process to avoid breaking the blinding. Research nurses, who operated the infusion, did not take part in the evaluation process in the trial. Besides, simulation agent and Oseltamivir had an identical appearance and taste. Simulation agent of Oseltamivir was made by Jiangsu Kangyuan pharmaceutical co. LTD, which did the blinding test in accordance with the drug quality standard approved by CFDA and issued the test report.

### Clinical monitoring

Researchers accessed and recorded the severity score of 8 influenza symptoms of patients at baseline (before treatment), 1 d, 2 d, 3 d after treatment, and the last day (end of all the treatment). A 4-level score was applied in accessing the severity of every symptom: fever (0,< 37.2°C; 3, 37.3˜37.9°C; 6, 38.5˜38.9°C; 9, above 39°C); aversion to cold and myalgia (0, absent; 2, mild; 4, moderate; 6, severe); cough, nasal obstruction, sore throat, fatigue, and headache (0, absent; 1, mild; 2, moderate; 3, severe).

Axillary temperature was taken by patients with a digital thermometer and recorded in patient diary card during the study, 10 times on day 1 (0.5 h, 1 h, 1.5 h, 2 h, 3 h, 4 h, 6 h, 8 h, 10 h, 12 h after treatment), and 6 fixed times on day 2 to day 5 (8:00, 10:00, 14:00, 16:00, 20:00, 22:00).

### Outcome measurements

### Primary outcome

The first primary outcome is the median fever alleviation time, which was measured in hours. The fever alleviation time was defined as time from baseline to the first time when axillary temperature descended more than 0.5°C.

Another primary outcome is the median fever clearance time, which was measured in hours. The fever clearance time was defined as time from baseline to the first time when axillary temperature decreased to < 37.4°C and maintenance of stable temperature (< 37.4°C) more than 24 hours.

### Secondary outcome

Three secondary endpoints were analyzed. These were the total 8 influenza symptom scores, the single influenza symptom score, and the frequency of aspirin usage.

### Safety evaluation

Safety was evaluated using vital signs, adverse reactions, electrocardiography, and clinical laboratory tests. These indices were compared before and after the using of test drugs.

### Statistical analyses

SAS (version 6.0) software (Statistical Analysis System, SAS Institute, Cary, NC, USA) was used for all the statistical analyses.

The primary outcomes were carried out for patients who completed study and had laboratory confirmed influenza infection. The secondary outcomes analysis were performed for all subjects who received study drug. Patients who received at least one time drug were included in the safety assessment.

The fever alleviation and clearance time were expressed as P50, using univariate COX regression model comprising time-censored data to analyze the differences between two groups. Total influenza symptom scores between groups were described using a *t*-test or *t*’test (if variance is absent). Single influenza symptom score between groups were described using the two independent sample Wilcoxon rank sum test. Comparison of total influenza symptom scores in group itself before and after treatment, paired-sample *t* test was applied. Comparison of single influenza symptom scores in group itself before and after treatment, the two related sample Wilcoxon rank sum test was applied. A comparison between usage rate of other treatment drugs for alleviating fever was undertaken using the *X*^2^ test.
